# Microbial Cell-Free DNA Sequencing in the Identification of Rhizopus Arrhizus in an Adolescent With Gastrointestinal Mucormycosis

**DOI:** 10.7759/cureus.97426

**Published:** 2025-11-21

**Authors:** Bibi Patel, Vini Vijayan, Kevin Tran

**Affiliations:** 1 Pediatrics, Valley Children’s Healthcare, Madera, USA

**Keywords:** fungal infection, karius test, microbial cell-free dna sequencing, mucormycosis, rhizopus, san joaquin valley

## Abstract

Mucormycosis is a rare and often fatal angioinvasive fungal infection, typically occurring in immunocompromised hosts. Gastrointestinal mucormycosis (GM), a rare manifestation of mucormycosis, is particularly challenging to diagnose due to its non-specific clinical presentation and overlap with other abdominal pathologies. We report the case of a previously healthy 15-year-old male with Class I obesity who presented with perforated appendicitis and developed septic shock and multi-organ failure for which he was admitted to the pediatric intensive care unit. Upon arrival, the patient was intubated and placed on mechanical ventilation. He was started on vasopressor support and intravenous antibiotics. An orogastric tube was placed for bowel decompression, but on hospital day 2, he developed profuse bloody output from the orogastric tube. Imaging revealed mixed-density debris surrounding the gastric lumen. His condition deteriorated with ongoing fevers and signs of gastrointestinal bleeding. Conventional infectious disease tests were unrevealing. Metagenomic next-generation sequencing of plasma cell-free DNA (Karius test) detected *Rhizopus arrhizus*, leading to the initiation of antifungal therapy with liposomal amphotericin B and posaconazole. Exploratory laparotomy revealed necrotic ulcers, extensive gastric and bowel necrosis and infarctions, confirming GM. Despite aggressive antifungal treatment and surgical debridement, the patient ultimately succumbed to his disease. This case underscores the importance of considering mucormycosis in critically ill patients without traditional risk factors and highlights the utility of microbial cell-free DNA sequencing as a non-invasive diagnostic adjunct in children with mucormycosis when tissue sampling is impractical.

## Introduction

Mucormycosis is a rare, opportunistic angioinvasive fungal infection caused by fungi of the order Mucorales, with *Rhizopus, Mucor, *and* Lichtheimia* accounting for an estimated 70%-80% of all cases [1]. Since the onset of the COVID-19 pandemic, there has been a significant rise in cases of mucormycosis globally, including a 2.1-fold increase in reported cases, with India alone documenting over 47,000 cases in 2021 [2]. Although these fungi are ubiquitous in nature, infection is associated with significant morbidity and mortality, particularly in patients with underlying risk factors such as diabetes mellitus, hematologic malignancies, prolonged immunosuppression, iron overload, trauma, or extensive burns. Mucormycosis can affect any organ system, but the most frequently affected sites include the rhino-orbital-cerebral, pulmonary, cutaneous, and gastrointestinal tracts [1,3].

Gastrointestinal mucormycosis (GM) is the rarest form and comprises only 4%-7% of all systemic mucormycosis cases, with mortality rates approaching 85% [3,4]. The stomach is the most commonly involved organ, followed by the colon and the ileum [4]. Infection occurs when Mucorales spores gain entry into a susceptible host through ingestion, inhalation, or introduction via contaminated food or drinks, environmental sources, or medical devices [4,5]. Following spore ingestion or mucosal translocation, the organism causes angioinvasion. This can lead to ischemia, tissue necrosis, gastrointestinal perforation, hemorrhage, and rapid clinical deterioration. Although GM has traditionally been associated with immunocompromised states, cases have increasingly been reported in patients with transient immune dysregulation, premature neonates, and even in otherwise healthy individuals without significant risk factors [3-6]. Early diagnosis is critical, as delays in recognition and treatment of mucormycosis are associated with markedly increased mortality. Studies suggest that postponing appropriate antifungal therapy by even six days can significantly worsen outcomes due to the infection’s rapid angioinvasive progression [1].

This case report describes a previously healthy 15-year-old adolescent with Class I obesity who initially presented with perforated appendicitis, subsequently developed gastrointestinal hemorrhage, and was later found to have GM. Diagnosis was established through the Karius test, a microbial cell-free DNA (cfDNA) sequencing assay, highlighting the potential utility of molecular diagnostics in identifying mucormycosis in critically ill patients without classic predisposing conditions.

## Case presentation

**Figure 1:**
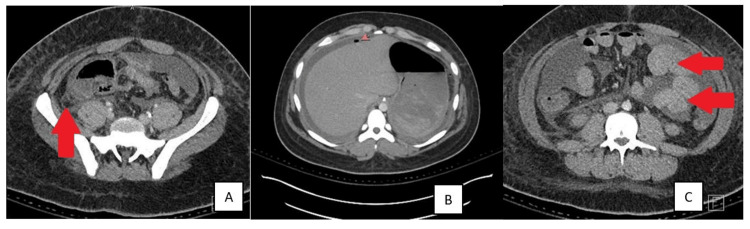
A computed tomography scan of the abdomen and pelvis demonstrated perforated appendicitis [A] with pneumoperitoneum [B] and multiple intra-abdominal fluid collections [C], the largest of which was located in the right upper quadrant.

A 15-year-old Hispanic male with a past medical history of Class I obesity presented to an outside facility with four days of progressive gastrointestinal symptoms, including intermittent, non-bilious, non-projectile vomiting occurring 3-4 times per day, persistent fever, and worsening, intermittent, diffuse, cramp-like abdominal pain that became constant. His symptoms intensified over the 48 hours before presentation, prompting evaluation in the emergency department. A computed tomography scan of the abdomen and pelvis demonstrated perforated appendicitis with multiple intra-abdominal fluid collections, the largest of which was located in the right upper quadrant (Figure 1).

He subsequently developed septic shock with multi-organ failure and was transferred to our pediatric intensive care unit for a higher level of care. Upon arrival, the patient was intubated and placed on mechanical ventilation. He was started on vasopressor support for hypotension and broad-spectrum intravenous antibiotics. He underwent bowel decompression soon after admission.

**Figure 2:**
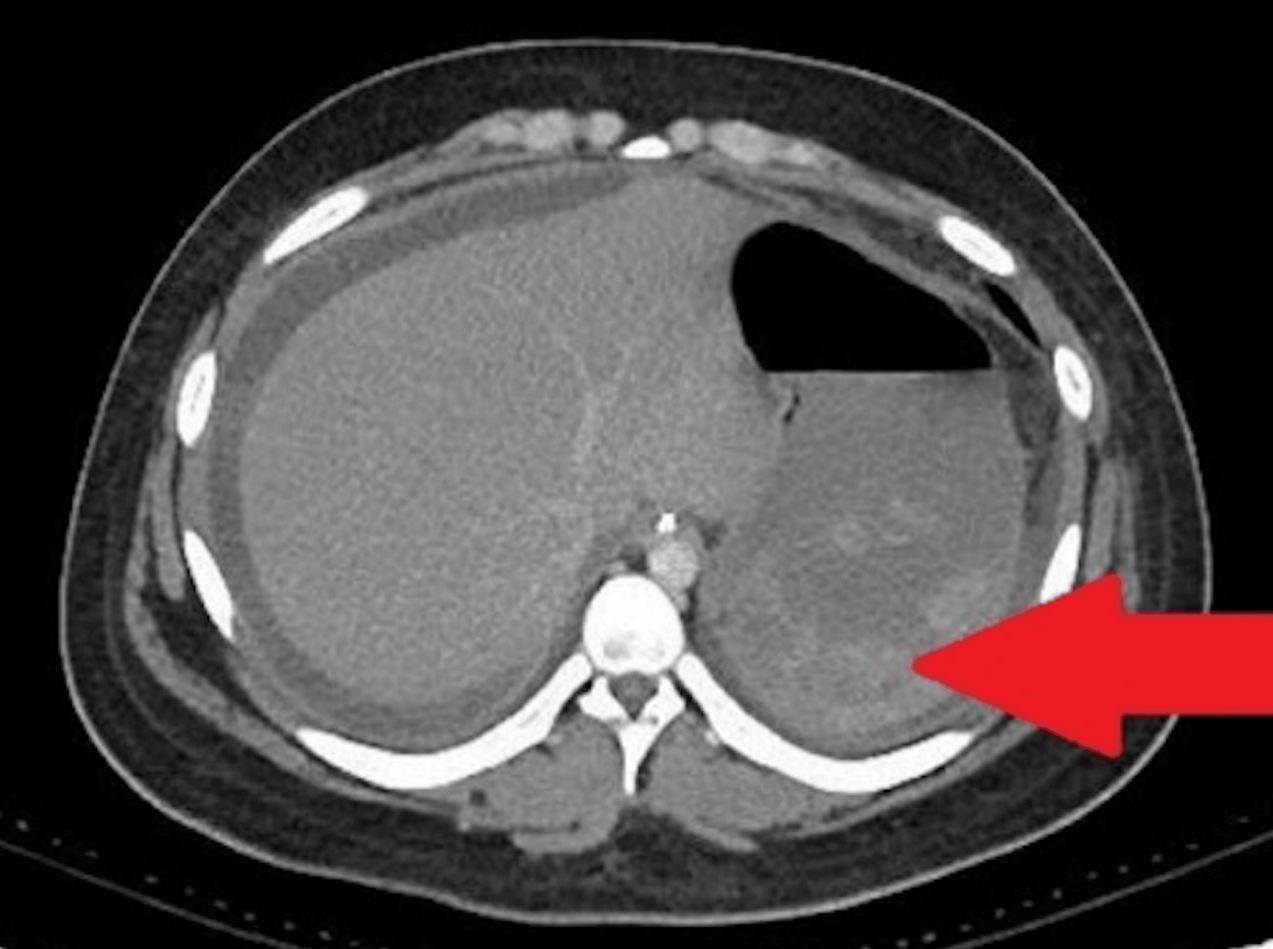
CT abdomen showing mixed echogenic debris in the gastric lumen. CT, computed tomography.

On hospital day 2, the child had 400 mL of bloody output from his orogastric tube. Repeat imaging revealed mixed-density debris surrounding the gastric lumen (Figure 2).

Laboratory evaluation demonstrated thrombocytopenia, coagulopathy, anemia, indirect hyperbilirubinemia, elevated lactate dehydrogenase, and acute renal failure (Table 1).

**Table 1:** Laboratory findings aPTT, activated partial thromboplastin time; BUN, blood urea nitrogen; INR, international normalized ratio; LDH, lactate dehydrogenase.

Test	Patient value	Reference range
Hemoglobin (g/dL)	7.9	13.0–16.0
Platelet count (×10³/µL)	59	150–450
Prothrombin time (sec)	19.9	11–15
INR	1.7	0.8–1.2
aPTT (sec)	47.9	25–35
Indirect bilirubin (mg/dL)	1.1	0.1–0.8
LDH (U/L)	518	100–190
BUN (mg/dL)	104	7–20
Creatinine (mg/dL)	4.8	0.5–1.0

These findings were suggestive of a thrombotic microangiopathic process, prompting evaluation for hemolytic uremic syndrome and thrombotic thrombocytopenic purpura, which were negative. A comprehensive infectious evaluation was performed, including serial blood cultures, stool bacterial and viral pathogen panels, and serum testing for cytomegalovirus, Epstein-Barr virus, human immunodeficiency virus, and adenovirus, all of which were negative. Fungal blood cultures, serumβ-D-glucan*,* and galactomannan assays were obtained and were negative. Despite supportive care with transfusions of packed red blood cells, platelets, and plasma, his clinical status continued to decline. Persistent fevers prompted a more extensive workup, including microbial cfDNA sequencing (Karius), which identified* Rhizopus arrhizus.*

**Figure 3:**
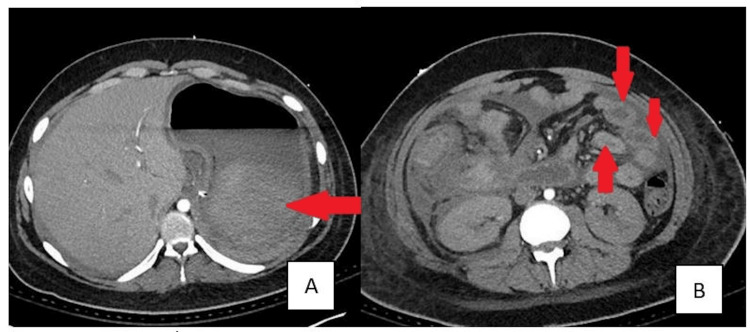
CT abdomen depicting the worsening of mixed-density debris in the gastric lumen [A] and multifocal abscesses [B]. CT, computed tomography.

The patient was promptly started on liposomal amphotericin B and posaconazole for antifungal coverage. Repeat imaging revealed worsening of mixed-density debris in the gastric lumen along with new multifocal abscesses with multiple areas of interloop abscesses (Figure 3).

An exploratory laparotomy was performed, revealing extensive gastric and bowel necrosis, necessitating aggressive surgical debridement. Immunologic evaluation, including HIV testing and hemoglobin A1c, yielded normal results, ruling out underlying immunodeficiency or diabetes. Despite maximal antifungal therapy and repeated surgical interventions, the patient's condition worsened, with ongoing hypotension, hemorrhage, and progressive multi-organ failure. He ultimately succumbed to his disease. Based on clinical presentation, surgical findings, and microbiologic confirmation, he was diagnosed with GM due to *R. arrhizus.*

## Discussion

The diagnosis of GM remains particularly challenging and is often delayed due to its non-specific clinical manifestations, the limited sensitivity of conventional diagnostic modalities, and the common misconception that it occurs exclusively in immunocompromised hosts. This case underscores the importance of maintaining a high index of suspicion for GM in patients presenting with unexplained gastrointestinal bleeding or necrosis, even in the absence of overt immunosuppression, and the need to consider molecular diagnostics early in the disease course to allow for targeted antifungal therapy.

GM is exceptionally rare, especially in immunocompetent hosts [1,3]. In a review by Kaur et al. [4], 176 cases of GM in immunocompetent patients were identified over the course of 69 years. The majority were from Asia, and the incidence was nearly equal in pediatric and adult patients. Among the pediatric patients, malnutrition and prematurity were noted to be the most common predisposing factors. Our patient had no known immunodeficiency, and immunologic testing, including HIV and hemoglobin A1c, was unremarkable. His only underlying condition was obesity, which has not been firmly established as an independent risk factor for mucormycosis. However, chronic low-grade inflammation, impaired innate immunity, and gut microbiome dysbiosis observed in obesity may predispose critically ill individuals to opportunistic infections, including invasive fungi [6]. Additionally, although the patient did not have traditional chronic risk factors for mucormycosis, several acute physiologic and treatment-related factors likely contributed to susceptibility. His perforated appendicitis resulted in bacterial peritonitis, gut barrier disruption, and profound systemic inflammation, all of which can facilitate translocation of Mucorales spores across compromised gastrointestinal mucosa. In addition, septic shock and multi-organ failure may have caused transient immune dysregulation. The patient also received broad-spectrum antibiotics and likely corticosteroids as part of standard septic shock management, both of which can alter innate immune function and further increase vulnerability to opportunistic fungi. Collectively, these factors provide a plausible explanation for the development of invasive mucormycosis in an otherwise healthy adolescent.

Our case demonstrates that although rare, GM should be considered in immunocompetent patients to enable prompt surgical debridement and initiation of antifungal therapy.

The clinical presentation of GM is non-specific, typically including abdominal pain, nausea, vomiting, gastrointestinal bleeding, or signs of sepsis [4,7-10]. GM can lead to ulcerative lesions and perforation, with extension resulting in bowel infarction, peritonitis, and hemorrhagic shock. Imaging features are similarly ambiguous, often demonstrating bowel wall thickening, perforation, or fluid collections, findings that overlap with other intra-abdominal pathologies. Consequently, GM frequently remains undiagnosed until surgical exploration or postmortem examination [7-10]. In a study by Antony et al. [7], 14 cases of GM in immunocompetent adults were described. Gastrointestinal bleeding was a presenting sign, and rapid deterioration following the bleed was noted in nearly all the patients, similar to the presentation and clinical course observed in our patient. The causal relationship between the patient’s perforated appendicitis and the development of GM remains uncertain. Histopathologic evaluation of the appendix was not available, limiting the ability to determine whether fungal invasion preceded appendiceal perforation. However, the clinical course suggests that mucormycosis was more likely a secondary invasive infection arising in the context of severe intra-abdominal inflammation, bacterial peritonitis, and progressive multi-organ dysfunction rather than the primary cause of appendicitis.

Traditionally, the gold standard for diagnosing mucormycosis has been direct microscopy, fungal culture, and histopathology demonstrating broad, ribbon-like, non-septate hyphae with angioinvasion [1,11]. Serologic testing (e.g., lateral flow assays and enzyme immunoassays) may offer adjunctive utility but is limited by poor sensitivity in immunocompromised hosts and an inability to distinguish between past and current infection [1,11]. In this case, serum β-D-glucan and galactomannan assays were obtained and were negative, which is expected because Mucorales do not produce galactomannan and lack (1→3)-β-D-glucan in their cell walls. Additionally, the diagnostic yield of fungal cultures is low, and only ~50% of cases are culture-positive due to sampling limitations and the fastidious nature of Mucorales. Plasma microbial cfDNA sequencing has emerged as a promising tool for diagnosing invasive fungal infections. Zhang et al. [12] performed a retrospective study that analyzed 14 pediatric mucormycosis cases and demonstrated that metagenomic next-generation sequencing (mNGS) had significantly higher sensitivity than conventional diagnostic methods, detecting mucormycosis pathogens in all patients, even when cultures and histopathology failed. Most cases involved immunocompromised children with hematologic malignancies, but notably, one immunocompetent child was also affected, like our patient.

 In our case, the patient’s progressive clinical deterioration, persistent fevers, and an inconclusive conventional infectious workup prompted the use of microbial cfDNA sequencing via the Karius test, which identified *R. arrhizus*. To our knowledge, this represents the first reported case of GM diagnosed using cfDNA sequencing via the Karius platform. The identification of this pathogen allowed for the initiation of antifungal therapy.

Plasma microbial cfDNA sequencing offers distinct advantages due to its non-invasive specimen collection and broad-spectrum pathogen detection diagnostic approach that does not require prior pathogen suspicion. It also has a relatively rapid turnaround time and the ability to identify rare or unexpected organisms. Studies have demonstrated its ability to detect deep-seated and bloodstream infections more rapidly than traditional methods [12,13]. However, mNGS results must be interpreted with caution. The test can identify low-level or transient microbial DNA that may not be clinically relevant, and it does not provide antifungal susceptibility profiles. Additionally, cost and limited availability may hinder widespread adoption. These features are especially relevant in the care of critically ill patients, where diagnostic delays can be fatal. Importantly, histopathologic demonstration of tissue invasion remains the gold standard for “proven” mucormycosis, and plasma cfDNA sequencing should be interpreted as an adjunctive modality that supports but does not replace clinicopathologic correlation in establishing a probable diagnosis.

In this case, while we cannot definitively exclude the possibility that* R. arrhizus* represented colonization or an incidental finding, several factors support its pathogenic role: (1) the fulminant clinical course, (2) extensive necrotic gastric and bowel tissue requiring surgical debridement, (3) imaging consistent with severe invasive gastrointestinal disease, and (4) the absence of any other identified pathogens despite exhaustive testing. Collectively, these findings, together with the detection of* R. arrhizus* by plasma microbial cfDNA sequencing, support a probable diagnosis of GM.

This case contributes to the growing body of literature supporting cfDNA sequencing as a valuable adjunct in the diagnosis of invasive fungal disease, particularly in scenarios where conventional diagnostics fail or tissue sampling is not feasible. While early antifungal therapy and surgical debridement remain the cornerstone of mucormycosis treatment, novel diagnostics such as plasma cfDNA sequencing facilitate pathogen identification and guide timely clinical decision-making, a critical consideration in fulminant infections such as GM. In our case, while cfDNA testing facilitated pathogen identification, the patient already had irreversible bowel necrosis and multi-organ failure before antifungal initiation.

 Further studies are needed to identify high-yield patient populations, define the complementary role of cfDNA to conventional microbiological methods, and discern how best to integrate mNGS into current testing algorithms.

## Conclusions

This case highlights the importance of considering GM even among previously healthy or minimally immunocompromised patients who present with unexplained gastrointestinal bleeding, necrosis, or clinical deterioration. Although rare, GM carries a high mortality risk due to its angioinvasive nature, which can lead to infarction, bowel perforation, and hemorrhagic shock. Given its non-specific presentation and the fulminant course, a high index of clinical suspicion and prompt initiation of antifungal therapy are crucial for improving outcomes. In this critically ill patient, plasma microbial cfDNA sequencing supported a probable diagnosis of GM, facilitating antifungal treatment when conventional testing was non-diagnostic and histopathologic confirmation was not available. While cfDNA sequencing facilitated pathogen detection and guided antifungal therapy, the patient’s extensive gastrointestinal necrosis, multi-organ failure, and advanced disease stage ultimately limited the potential for clinical recovery.

This case underscores the potential utility of cfDNA sequencing as a complementary diagnostic tool in complex or rapidly progressive presentations where tissue sampling is not feasible. However, it also emphasizes the continued need for tissue-based confirmation whenever possible, as cfDNA alone cannot establish a definitive diagnosis. Further studies are needed to determine the clinical utility, cost-effectiveness, and optimal integration of mNGS into standardized diagnostic pathways for mucormycosis and other invasive fungal infections. Given the increasing global burden of mucormycosis, clinicians should maintain a high index of suspicion for GM in high-risk and rapidly deteriorating patients, regardless of immune status, and consider integrating advanced molecular diagnostics to facilitate early recognition and targeted treatment.
